# Impact of COVID-19 on laboratory professionals-A descriptive cross sectional survey at a clinical chemistry laboratory in a developing country

**DOI:** 10.1016/j.amsu.2020.07.022

**Published:** 2020-07-18

**Authors:** Lena Jafri, Sibtain Ahmed, Imran Siddiqui

**Affiliations:** Department of Pathology and Laboratory Medicine, Aga Khan University, Stadium Road, P.O. Box 3500, Karachi, 74800, Pakistan

**Keywords:** SARS-CoV, Impact, Laboratories, Surveys

## Abstract

**Background:**

The lab professionals at one end are at increased risk of contracting the infection while on the other end have to deal with the various challenges during the Coronavirus Disease 2019 (COVID-19) outbreak. This survey was undertaken to analyze the lab professionals' perspectives, in terms of the challenges, financial implications, fears, motivation and satisfaction from organizational processes and policies adopted, amid the COVID-19 crisis.

**Material & methods:**

The study utilized a cross-sectional survey design. The survey was administered online via the google docs survey tool from medical laboratory professionals (n = 64) serving at the section of Clinical Chemistry, department of Pathology and Laboratory Medicine, the Aga Khan University (AKU), Pakistan from June 4th to 14th 2020. A team consisting of three Clinical Chemistry consultants serving as faculty at the section, designed the survey questionnaire. The responses were recorded on a five-point Likert Scale (1 = strongly disagree, 2 = disagree, 3 = neutral, 4 = agree and 5 = strongly agree). The statistical analysis was performed using the Microsoft Excel 2013. Frequency and percentages were calculated for gender, experience and designation while descriptive results based on the responses were also recorded.

**Results:**

The response rate was 78% (n = 50). 60% responded that the current lifestyle adopted during the pandemic was not better than the traditional one. The fear of employment termination and financial challenges were being faced by 42% and 78% respondents respectively. The quality of family life was improved in 54% cases while 96% were of the view that their social activities at work have suffered. Whereas, 22% were not satisfied by the measures taking by the management during the outbreak.

**Conclusion:**

The findings of this survey provide laboratorians' perspective, in times of such crisis and provides us certain lessons to prepare for such unpredicted situations in future.

## Highlights

• 68 % lab staff was overall satisfied with the measures adopted by the institution to tackle the crisis.
• Majority of the respondents were in favor of the conventional pre COVID work environment.
• 44 % responded that there was inadequate and untimely provision of personal protective equipment.
• 34% of the lab professionals expressed that their motivation levels have gone down due to the challenges.

## 1 Introduction

A respiratory and systemic illness caused by severe acute respiratory syndrome coronavirus 2 (SARS-CoV-2), widely termed as Coronavirus Disease 2019 (COVID-19) emerged as a pandemic in the beginning of 2020 and has spread widely, according to the statistics reported by the world health organization (WHO) [1,2]. The COVID-19 has activated an unanticipated and unparalleled worldwide emergency, which has drastically impacted many organizations in terms of financial crisis and revenue generation, also including clinical laboratories [3,4].

The role of laboratory medicine in times of an infectious disease outbreak has been widely established [3]. Literature review contributes to the fact that the etiological diagnosis of COVID-19, was not possible without the laboratory services, either by detecting the pathogen in biological samples with reverse transcriptase-polymerase chain reaction (RT-PCR), or by quantifying antibody response immunologically [5]. From a laboratorian's perspective, there are several areas in which the clinical chemistry laboratories can contribute amid the COVID-19 crisis, ranging from contributions to establishing diagnosis, prognosis, disease staging, therapeutic drug monitoring and epidemiologic surveillance studies [6]. Owing to the global recession, for the past few years, clinical chemistry laboratory services worldwide have greatly suffered at the hands of hefty, repeated and usually unreasonable cost-cutting practices, leading to the misery and agony of the laboratory professionals [7].

In a developing country like Pakistan more than 90% of the clinical laboratories are small and without adequate physical and man power infrastructures [8]. Furthermore, working close to the “minimal level of compensation” has hence become routine in the vast majority of laboratory services in the country especially clinical chemistry, where employs repeatedly suffer layoffs due to the growing automation in the sector. The outbreak has no doubt, overburdened the already vulnerable laboratories with challenges such as issues related to availability of manpower, transportation issues, lack of personal protective equipment (PPE), delayed shipments of essential supplies, employs health, declining revenue targets and specially fear and anxiety amongst the frontline staff [9]. Despite all these challenges, laboratory medicine is again projecting its intrinsic and well-known suppleness, with the uninterrupted provision of diagnostic services 24/7 [5].

The Clinical Chemistry laboratory at the Aga Khan University Hospital (AKUH), Karachi, is the largest in the country and serves as a national referral center with more than 50 full time serving laboratory professionals and six consultant Chemical Pathologists. With its growing network of more than 250 phlebotomy stations and stat laboratories spread across Pakistan, it eventually caters to test requests from all the provinces [10]. The laboratory operates to highest standards of quality and was the first to be accredited by Joint Commission International Accreditation (JCIA) and the only with College of American Pathologist (CAP) accreditation in Pakistan. Medical laboratory professional, which includes pathologists, managers, technologists, and pathology residents, are critical members of the health care team. The Clinical Chemistry laboratory serves as a referral center, and caters to on an average 16000 routine and specialized tests requests per day from the entire country. The laboratory was the first to initiate diagnostic testing for COVID-19 suspected cases in the country. The clinical chemistry section, continued 24/7 operations even during the imposed lock down and provided diagnostic facilities to routine as well as COVID-19 cases. The preparations for such a crisis never existed in the books of laboratories especially for resource constrained setups in developing world, however the section undertook certain timely measures to ensure employs wellbeing and safety. As a result, none of our staff was tested positive during the outbreak, despite handling COVID positive samples.

The aim of this survey was to analyze the lab professionals' perspectives serving at the clinical chemistry laboratory, in terms of the challenges, financial implications, fears, motivation level and satisfaction from organizational processes and policies adopted, amid the COVID-19 crisis.

## 2 Methods

This study utilized a cross-sectional survey design and was conducted at the section of Clinical Chemistry, Department of Pathology and Laboratory Medicine, the Aga Khan University (AKU), Karachi, from June 4th to 14th 2020, when the local spread of the pandemic was on the rise in the country. The study was exempted from the institutional ethical review committee (ERC#2020-4861-10674). The survey was administered online via the *google docs* survey tool, to elicit information about social and financial well-being, stress due to COVID-19 pandemic, satisfaction with organizational policies and practices among medical laboratory professionals.

A team consisting of three Clinical Chemistry consultants serving as faculty at the section, designed the survey questionnaire with discussions on COVID-19 and its impact on personal and professional lives of laboratorians. With the laboratory staff's inherent time constraints in mind, the survey was structured in such a way that it did not consume more than 10 min of a participant's time. It consisted of 16 items grouped into two sections. The initial section consisted of demographics with a description of their role in the lab, experience and educational level. The next section was more oriented towards social, mental and financial impact of COVID-19 on the employees alongside their satisfaction and awareness from organizational policies, practices and measures taken amid the pandemic emergency, adequacy of resources and extent of training. To validate the questionnaire, it was initially filled as a pilot by one resident, one faculty member and one senior technologist.

The questionnaire was then self-administered using google docs online. An email requesting participation was sent to all the full-time lab professionals including technologists, managers, residents and faculty serving at the section of Clinical Chemistry (n = 64). The sample size was not predetermined, and a purposive approach of simultaneous data collection and analysis was taken until time saturation was reached based on the pre-set final date of attempting the survey. This has been included in the revised manuscript. A reminder email was sent two days after the initial request to ensure maximum participation. The Admin staff, support staff and trainee technologists were excluded as they were relieved from non-essential services during the outbreak. Additionally, the housekeeping staff was not included due to their limited ability to participate in an online survey.

Electronic consent for participation was acquired at the initial page of the survey. The participation in the survey was completely voluntarily and any person can opt out and withdraw by not submitting the answers. To ensure confidentiality any personal details including email ids that might lead to identification of the personnel were not acquired as part of the survey. Additionally, all data was stored in a password protected electronic format. The email circulated addressed the goals of the survey and the re-assurance regarding the maintenance of anonymity of responses. The study was registered with the Chinese Clinical Trial Registry (Registration No: ChiCTR2000033899). Moreover, this work has been undertaken and reported in line with the STROCSS criteria [11].

## 3 Statistical analysis

The responses were recorded on a five-point Likert scale (1 = strongly disagree, 2 = disagree, 3 = neutral, 4 = agree and 5 = strongly agree). The statistical analysis was performed using the Microsoft Excel 2013 and Statistical Package of Social Sciences (SPSS) version 19. Frequency and percentages were calculated for gender, experience level and designation. While, descriptive results based on the responses were also recorded. Moreover, two groups were created based on the experience levels at the current lab i.e. group I having experience of ≥10 years and group II with experience of <10 years respectively. A chi-square test of independence was performed to examine the relation between experience level and measures of financial challenges, motivation level, fears and satisfaction. Two-tailed p-values < 0.05 were considered significant.

## 4 Results

This survey presents data from fifty professionals at varying stages in their professional careers at the Clinical Chemistry section of the clinical laboratories of AKUH. After an informed consent, a total of 78% (n = 50) responses were received. Respondents were predominantly female. Table 1 presents the descriptive characteristics of the participants.

**Table 1:** Description of the participants (n = 50).

Variables		Number (n)	Parentage (%)
Gender	Female	30	60
	Male	20	40
Designation	Senior Technologists	17	34
	Technologists	15	30
	Charge Technologists	7	14
	Faculty & Managers	6	12
	Residents	3	6
	Coordinators	2	4
Education level	Masters of Science	36	72
	Bachelors of Science	6	12
	Fellowship in Clinical Chemistry from College of Physicians and Surgeons Pakistan (FCPS)	5	10
	Bachelors of Medicine & Surgery (MBBS)	3	6
Experience at the current laboratory	5–10 years	21	42
	10–20 years	12	24
	Less than 5 years	10	20
	More than 20 years	7	14

### 4.1 Social and financial well-being

Indicators of social and financial well-being included the respondent's quality of social life at home and work place and whether they were facing financial challenges during these tough times. These measures provide a bigger picture of how satisfied they are, how much they are enjoying their work and their perceptions of the adequacy of their compensation. Majority of the respondents i.e. 60% (n = 30) believed that the current life style adopted during the pandemic of social distancing, virtual meetings and remote functioning was not better than the traditional, pre COVID 19 times.

**Fig. 1:**
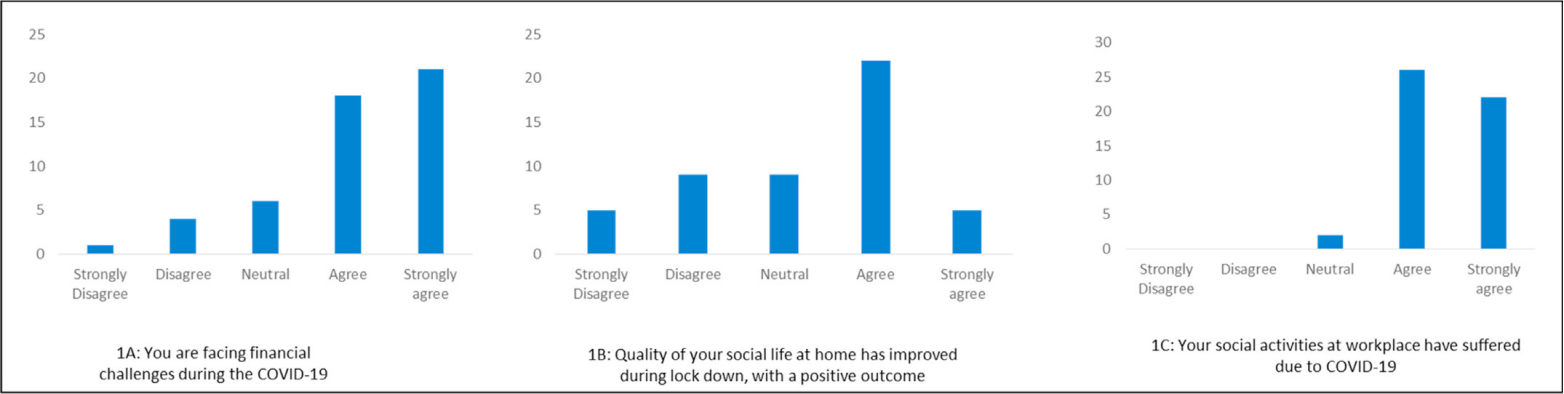
Financial and social implications.

More than 78% (n = 39) of the staff members were facing financial challenges during the crisis. Approximately 54% (n = 27) responded that the quality of your social life at home has improved during the lockdown, with a positive outcome on their wellbeing. Whereas, more than 96% (n = 48) agreed that their job environment has suffered significantly in terms of social activities at work. Furthermore, the range of information gathered from this survey, focusing on the social and financial consequences rated on the Likert scale are summarized in Fig. 1.

### 4.2 Stress due to COVID-19 pandemic

To ensure staff's sense of meaning and engagement indicators of stress included in the survey were an inquiry regarding their motivation level amidst the COVID-19 times and the impact of declining workload on their routine activities. More than half i.e. 62% (n = 31) of the laboratorians agreed that they feel the same level of energy and enthusiasm while coming to work, which they had prior to these rough times. Furthermore, a noteworthy factor is quality control activities and accreditation requirement which serves as a constant source of stress for the lab professionals. However, 80% (n = 40) respondents agreed that the decreasing workload has provided them enough time to focus on quality control and assurance activities including accreditation requirements and documentation tasks.

**Fig. 2:**
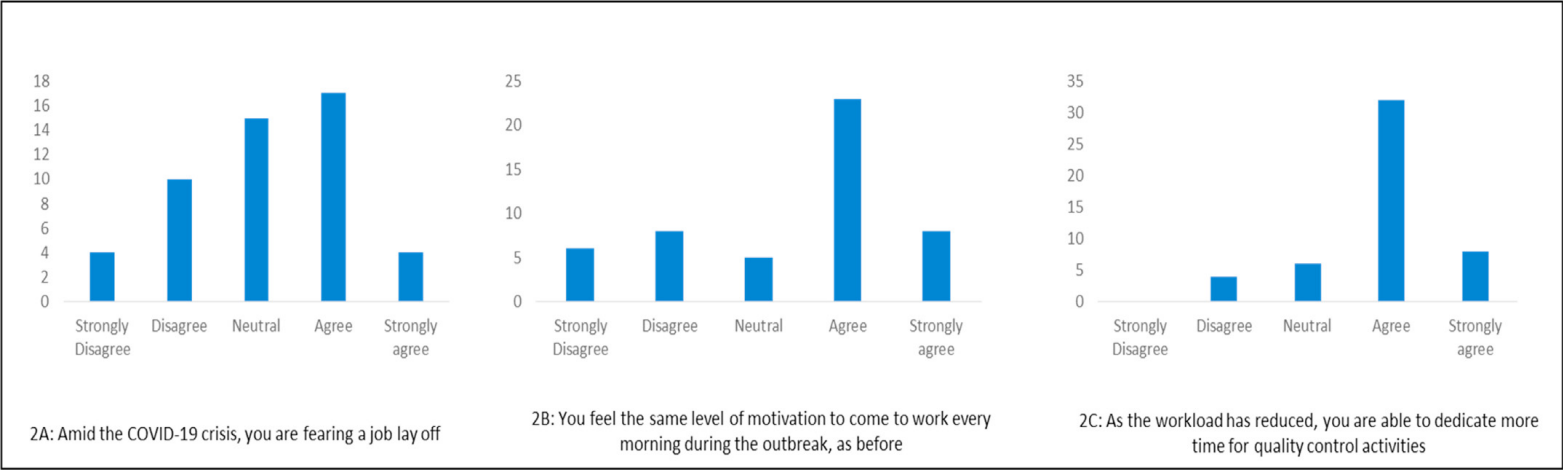
Indicators of stress due to COVID-19 pandemic.

Only 8% (n = 4) lab professionals strongly agreed and 34% (n = 17) agreed to the fact that an overhanging sword of employment cut exists during the COVID crisis and were constantly fearing a lay off if the current financial crunch prolongs, despite repeated re-assurance from the administration. Furthermore, the information elicited focusing on elements of stress are depicted in Fig. 2.

### 4.3 Satisfaction with organizational policies and practices

**Fig. 3:**
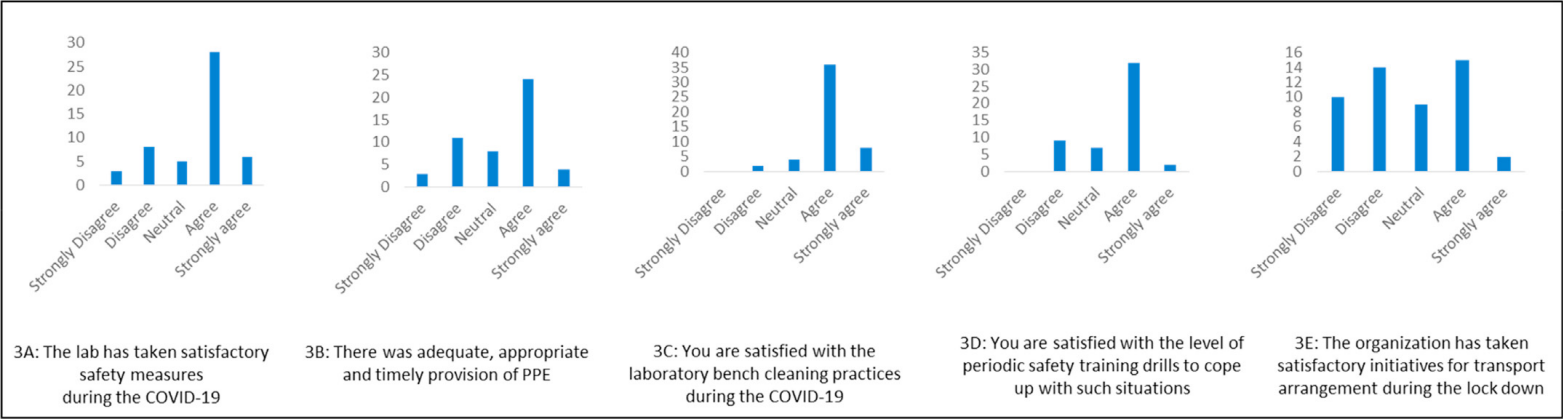
Satisfaction from policies and practices.

Only 50% (n = 25) were aware that all staff and their dependents, in case, if tested positive, their complete care will be covered by the hospital, the laboratory is affiliated with, contrary to the routine practice in which the hospital bears 85% of the total expenditure of medical care provided. Additionally, information was collected regarding their overall contentment with the institutional policies undertaken during the outbreak including provision of personal protective equipment (PPE), cleaning practices and transportation arrangements alongside prior educational training to face such challenges. Majority of the participants i.e. 68% (n = 34) were overall satisfied with the measures adopted by the institution to tackle the crisis situation. Timely, appropriate and adequate provision of PPE was a major challenge due to shortage of supply and as anticipated only 56% (n = 28) respondents were satisfied to its delivery. As the housekeeping cleaning schedules were re-defined and the entire premises including the benches were cleaned several times a day with standard disinfection practices and 88% (n = 44) of the respondents were satisfied. Apart from 7 neutral responses, 18% (n = 9) of the staff believed that their prior safety training and education was lacking to effectively confront this situation. The organization has implemented transportation facilities for employs to use during the imposed lock down but only 34% (n = 17) of the staff were of the view that they were adequate and fulfilling their needs. The indicators of satisfaction from practices and policies are illustrated in Fig. 3.

Furthermore, a chi-square test of independence showed that there was no significant association between experience level and measures of financial challenges, motivation level, fears and satisfaction from organizational policies adopted amid the COVID times as shown in Table 2.

**Table 2:** Measures of financial challenges, motivation level, fears and satisfaction between the two groups categorized on the basis of experience level (n = 50).

	Group I (Experience at current lab ≥ 10 years)	Group II (Experience at current lab < 10 years)	*p-value*
You are facing financial challenges during the COVID-19	Agree = 9	Agree = 9	*p*= *0.358*
	Disagree = 1	Disagree = 3	
	Neutral = 1	Neutral = 5	
	Strongly agree = 7	Strongly agree = 14	
	Strongly disagree = 1	Strongly disagree = 0	
You feel the same level of motivation to come to work every morning during the outbreak, as before	Agree = 13	Agree = 10	*p*= *0.070*
	Disagree = 1	Disagree = 7	
	Neutral = 0	Neutral = 5	
	Strongly agree = 3	Strongly agree = 5	
	Strongly disagree = 2	Strongly disagree = 4	
Amid the COVID-19 crisis, you are fearing a job lay off	Agree = 6	Agree = 11	*p*= *0.293*
	Disagree = 6	Disagree = 4	
	Neutral = 5	Neutral = 10	
	Strongly agree = 0	Strongly agree = 4	
	Strongly disagree = 2	Strongly disagree = 2	
The lab has taken satisfactory safety measures during the COVID-19	Agree = 10	Agree = 18	*p*= *0.097*
	Disagree = 6	Disagree = 2	
	Neutral = 0	Neutral = 5	
	Strongly agree = 2	Strongly agree = 4	
	Strongly Disagree = 1	Strongly Disagree = 2	

## 5 Discussion

The human medical knowledge of disease diagnosis and management have conquered various horizons however, COVID-19 exposed our weakness and limitations. This study explored the impact of COVID-19 pandemic on the laboratorians in Pakistan. The rational being dire need of identifying the factors that affect laboratory professionals during such pandemic outbreaks. Various studies globally have explored the impact on the physical, mental and social well-being of health professionals but studies focused on clinical laboratory professionals are scarce**.** With this motive in context, a well-structured survey, can serve as a valuable tool harvesting a high rate of participation and valid database to establish correlations and/or associations between policies and practices alongside employ engagement during the COVID-19 [12,13]. As these circumstances were unprecedented and no prior valid questionnaire existed, the survey was conducted using a self-designed questionnaire keeping in view the needs of time, based on input from experienced clinical chemistry consultants, serving as faculty.

The response rate being 78% was acceptable. As anticipated most the of the respondent did not find the workplace life style adopted during the outbreak and were in favor of the earlier more traditional and social work place rituals. As clinical laboratories have already adopted cost saving practices in the pre COVID-19 days due to inflation, rising exchange rates and economic crisis in the country haling new hiring's and various financial incentives. The rate of respondents expressing concerns of being laid off from their positions, amidst the COVID-19, were in concordance with global literature [14].

Furthermore, the lockdown imposed in the country and the declining level of social activities lead to deterioration of mental health and increasing stress levels [15]. The sanctions imposed by the organization including 10–30% pay reduction based on seniority levels and abandonment of all appraisals in the fiscal year further hiked their agonies as 78% of the staff mentioned that they were suffering from financial challenges during the outbreak. Moreover, more than 34% of the employees felt that their motivation levels have gone down due to the implications, despite, timely and repeated re-assurance from their senior management.

Additionally, there were potential safety concerns as well regarding contacting the infection, as 44% responded felt that there was inadequate and untimely provision of PPE, amid short supplies and cost saving practices limiting their usage. Whereas, 22% were not satisfied by the measures taking by the lab management during the outbreak, the foremost being lack of daily temperature monitoring of lab staff. Most felt substantial mental stress as they too have families, and were naturally fearful that they might carry the virus home. Furthermore, as this crisis situation was unforeseen, the challenges and fears were experienced by the junior as well as well as the senior professionals, with no significant difference in responses between the two groups.

The lab management had come up with an ad hoc rescheduling of bench cleaning and housekeeping practices during the outbreak [16]. High exposure areas including the benches, tables etc. were cleaned and wiped down several times a day with 5% Hypochlorite and alcohol-based sanitizers as appropriate. The premises were mopped three times a day with disinfectant. Discarded PPE and other clinical waste were collected in biohazard bags and more frequently collected by the designated personnel and disposed in a proper manner. Due to the considerable measures adopted, more than 90% of the staff were satisfied with the activities as shown in Fig. 2C.

Furthermore, the imposed lock down by the government led to closure of public transport utilized by most of the lab staff for commuting to and fro from work. Additionally, pillion-riding on motorbikes was banned in Karachi and as a consequence female staff, which do not drive motorbikes in Pakistan normally, had to suffer as they were unable to share the ride with their male family members. The organization did provide transport service but they were inadequate, not covering majority of locations, untimely and eventually 66% of the lab professionals, majority being females, were not satisfied.

Web based surveys offer certain advantages such as no material cost, self-administration, rapid turnaround and a high degree of participation [17,18]. There were certain limitations of our study. Web-based surveys can be affected by issues pertaining to non-response and measurement error. Nonresponse error refers to the fact that not all lab professionals are ready and able to participate in the survey. Literature review have shown that email based online surveys often are not able to match the response rates of conventional mail surveys as stirring tactics, such as request letters, personalized signatures and individual letterhead are missing [19]. We administered the survey during COVID-19 outbreak; a few employs were relieved utilizing their annual earned leaves to prevent exposure with limited access to emails which may have negatively impacted response rate. Finally, measurement error could arise as already disturbed lab professionals may have lacked motivation, intentionally provided inaccurate information or not copiously comprehended the survey. The results are limited as all study variables were measured through self-reports from a single Clinical Chemistry laboratory and further research is needed to address these issues using multiple sources of information for a thorough comparison. The profession is quite diverse and the sample size of those surveyed was relatively small for some occupational groups. Nevertheless, to the best of our knowledge, this survey is the first to address clinical chemistry lab professionals' perspectives amid the COVID-19 calamity, thus the sample represents an important contribution to the field. Furthermore, eliciting staff concerns in times of the crisis to make them feel engaged and valued, leads to the flourishment of a trustworthy environment between the leadership and the lab professionals.

## 6 Conclusion

Like many other businesses at this point in time, laboratory professionals also continue to suffer from unprecedented challenges and fears. The financial implications, the declining motivation levels, inadequate provision of PPE harnessing safety concerns, additional stress of transport arrangement during the lock down and the fear of being laid off from the job amid the COVID-19 crisis are major concerns requiring immediate attention by the management, as they can potentially affect efficiency and productivity. It is too early to predict when this pandemic will terminate and the unforeseen consequences that might continue from then, the findings of this survey provides us certain lessons to prepare for such unpredicted crisis in times to come.

## Ethical approval

The study was granted exemption from the institutional ethical review committee of the Aga Khan University, Karachi (ERC#2020-4861-10674).

## Funding

None.

## Author contribution

Lena Jafri assisted with data collection and did majority of the write-up of the first draft. Sibtain Ahmed planned and designed the survey, performed the literature search, data collection and analysis and contributed with the write-up. Imran Siddiqui conceived the idea and coordinated the writing of the paper and reviewed the final draft. All the authors have accepted responsibility for the entire content of this submitted manuscript and approved submission.

## Registration of research studies

1. Name of the registry: Chinese Clinical Trial Registry
2. Unique Identifying number or registration ID: ChiCTR2000033899;
3. Hyperlink to your specific registration (must be publicly accessible and will be checked): http://www.chictr.org.cn/searchprojen.aspx?title=&officialname=&subjectid=&secondaryid=&applier=&studyleader=&ethicalcommitteesanction=&sponsor=&studyailment=&studyailmentcode=&studytype=0&studystage=0&studydesign=0&minstudyexecutetime=&maxstudyexecutetime=&recruitmentstatus=0&gender=0&agreetosign=&secsponsor=&regno=ChiCTR2000033899&regstatus=0&country=&province=&city=&institution=&institutionlevel=&measure=&intercode=&sourceofspends=&createyear=0&isuploadrf=&whetherpublic=&btngo=btn&verifycode=&page=1

## Guarantor

Dr. Sibtain Ahmed, Senior Instructor, Department of Pathology and Laboratory Medicine, Aga Khan University Hospital, Stadium Road, Karachi, Pakistan. P.O. Box 3500. Telephone: 021-34861927, Email: sibtain.ahmed@aku.edu.

## Provenance and peer review

Not commissioned, externally peer reviewed.

## Consent

Electronic consent for participation was acquired at the initial page of the online survey.

## Declaration of competing interest

None.
