# Supplementary material for: Impact of COVID-19 on laboratory professionals-A descriptive cross sectional survey at a clinical chemistry laboratory in a developing country
Source: Ann Med Surg (Lond). 2020 Jul 18;57:70–5. doi: 10.1016/j.amsu.2020.07.022 (PMC7367796; doi:10.1016/j.amsu.2020.07.022)
Supplement: Multimedia component 2 [file mmc2.docx]

**Appendix: Survey Questionnaire**

| **Current Designation** | | | | | | |
| --- | --- | --- | --- | --- | --- | --- |
| **Gender** | | | | | | |
| **Education level** | | | | | | |
| **Experience at this institution** | | | | | | |
| **If you or your dependents are tested positive for COVID-19, AKUH will provide medical care**  **(Tick one):** | | O Completely free of charge  O Employer will bear 85% of the expenditure  O Don’t know | | | | |
| **Do you think that the current life style (social distancing, virtual meetings) is better than the previous pre-COVID 19 days** | | O Yes  O No | | | | |
| **S. No** | **Question** | **Strongly**  **Disagree** | **Disagree** | **Neutral** | **Agree** | **Strongly**  **Disagree** |
|  |  | **1** | **2** | **3** | **4** | **5** |
| **1** | **Amid the COVID-19 crisis, you are fearing a job lay off** |  |  |  |  |  |
| **2** | **You are facing financial challenges during the COVID-19** |  |  |  |  |  |
| **3** | **Quality of your social life at home has improved during lockdown, with a positive outcome** |  |  |  |  |  |
| **4** | **Your social activities (meetings, break time, human interaction) at workplace have suffered due to COVID-19** |  |  |  |  |  |
| **5** | **You feel the same level of motivation to come to work every morning during the outbreak, as before** |  |  |  |  |  |
| **6** | **As the workload has reduced, you are able to dedicate more time for quality control activities** |  |  |  |  |  |
| **7** | **Our lab has taken satisfactory safety measures during the COVID-19** |  |  |  |  |  |
| **8** | **There was adequate, appropriate and timely provision of PPE** |  |  |  |  |  |
| **9** | **You are satisfied with the laboratory bench cleaning practices during the COVID-19** |  |  |  |  |  |
| **10** | **You are satisfied with the level of periodic safety training drills to cope up with such situations** |  |  |  |  |  |
| **11** | **The organization has taken satisfactory initiatives for transport arrangements during the lock down** |  |  |  |  |  |
